# Prediction of compressibility parameters of the soils using artificial neural network

**DOI:** 10.1186/s40064-016-3494-5

**Published:** 2016-10-18

**Authors:** T. Fikret Kurnaz, Ugur Dagdeviren, Murat Yildiz, Ozhan Ozkan

**Affiliations:** 1 Department of Geophysical Engineering, Engineering Faculty, Sakarya University, Serdivan, Sakarya Turkey; 2 Department of Civil Engineering, Engineering Faculty, Dumlupinar University, Kutahya, Turkey; 3 Department of Electrical and Electronics Engineering, Engineering Faculty, Sakarya University, Serdivan, Sakarya Turkey

**Keywords:** Artificial neural network, Compressibility, Compression index, Consolidation, Recompression index

## Abstract

The compression index and recompression index are one of the important compressibility parameters to determine the settlement calculation for fine-grained soil layers. These parameters can be determined by carrying out laboratory oedometer test on undisturbed samples; however, the test is quite time-consuming and expensive. Therefore, many empirical formulas based on regression analysis have been presented to estimate the compressibility parameters using soil index properties. In this paper, an artificial neural network (ANN) model is suggested for prediction of compressibility parameters from basic soil properties. For this purpose, the input parameters are selected as the natural water content, initial void ratio, liquid limit and plasticity index. In this model, two output parameters, including compression index and recompression index, are predicted in a combined network structure. As the result of the study, proposed ANN model is successful for the prediction of the compression index, however the predicted recompression index values are not satisfying compared to the compression index.

## Background

It is necessary to determine the compressibility parameters of soils such as the compression index (C_c_) and the recompression index (C_r_) for safe and economic design of civil engineering structures. In order to calculate the consolidation settlement of normally consolidated and over-consolidated saturated fine-grained soils, the compressibility parameters are determined by means of laboratory oedometer test on undisturbed samples based on Terzaghi’s consolidation theory. These parameters can be influenced from the quality of samples used in the tests. Although the compressibility parameters must be obtained from careful oedometer test measurements based on good quality undisturbed samples, conventional oedometer test comprises major disadvantages such as costliness, unwieldiness and time-consuming. In addition, the other important disadvantage of the estimation of the compressibility parameters is that the graphical method directly depends on the personal experience. Because of these factors, many researchers have been tried to develop practical and fast solutions.

The presence of relationships between the compressibility parameters and the basic soil properties has been investigated from past to present. Many different correlations based on multiple linear regression analysis have been proposed for determination of compression index (C_c_) soil by researchers (Skempton 1944; Terzaghi and Peck 1967; Azzouz et al. 1976; Nagaraj and Srinivasa Murthy 1985; Lav and Ansal 2001; Yoon et al. 2004; Solanki et al. 2008; Dipova and Cangir 2010; Bae and Heo 2011; Akayuli and Ofosu 2013; Lee et al. 2015). These studies are generally focused on relationships between the compression index and physical properties of the soils such as the initial void ratio (e_0_), natural water content (w_n_), liquid limit (LL), and plasticity index (PI). Besides, the studies on the recompression index (C_r_) seem to be quite limited (Nagaraj and Srinivasa Murthy 1985; Nakase et al. 1988; Isik 2009). The researches show that the physical parameters of soils have a significant effect on compressibility parameters of soil. In literature, the given regression equations to predict the compressibility parameters generally divided into two groups; connected with state variables such as void ratio and water content and connected with intrinsic variables such as liquid limit and plasticity index. It is known that the fully disturbed samples losing their memory involved with soil structure or stress history. Therefore, intrinsic properties are generally obtained by using fully disturbed samples. A number of previous researchers have reported that the compressibility of remolded clay has a specific relationship with the intrinsic variable of the clay (Lee et al. 2015). Due to the compression index of natural clay affected by the sedimentation case induced by deposition environments the evaluation using only intrinsic variables seems to be erroneous.

Artificial neural networks (ANNs) have become a commonly used method due to give a more efficient and accurate results according to regression based statistical equations especially in terms of making estimates in nonlinear systems. Recently, ANNs have been successfully applied to many geotechnical engineering problems such as slope stability, liquefaction, settlement behavior, bearing capacity of shallow and deep foundations (Lee and Lee 1996; Sakellariou and Ferentinou 2005; Kim and Kim 2006; Kuo et al. 2009; Kalinli et al. 2011; Sulewska 2011; Chik et al. 2014). Due to the compression index (C_c_) and the recompression index (C_r_) are affected by multiple parameters, many researchers have been used the soft computing methods to determine these indexes in a much shorter time (Isik 2009; Park and Lee 2011; Kalantary and Kordnaeij 2012; Namdarvand et al. 2013; Demir 2015).

In this paper, an ANN application was performed to determine the compressibility parameters by using the index parameters of fine grained soils as a variable. Thus, it was aimed to get much better results compared to the obtained by the empirical formulas based on the regression analysis in the previous studies. On the other hand, most of the other studies related to predict the compressibility parameters (compression or recompression index) by using ANN models have checked carefully and it was seen that these studies preferred single output models despite using a number of different input parameters. In the presented study, both the compression index and the recompression index were tried to predict by using a two-output combined ANN model based on natural water content (w_n_), initial void ratio (e_0_), liquid limit (LL) and plasticity index (PI). Two or more output models provided time saving and reduced the workload and also successful results have been obtained by these models. A total of 246 laboratory oedometer and index tests results of fine-grained soils obtained by the various geotechnical investigations in Turkey were used in this work. The performance of the proposed ANN model was evaluated based on the correlation coefficient (R) and mean squared error (MSE).

## Consolidation settlement

The settlement of structures on the fine-grained soil stratums due to the vertical stress increment is one of the most important problems in geotechnical engineering. The total settlement of a structure comprises three parts: (1) immediate (elastic) compression, (2) primary consolidation, (3) secondary consolidation (creep). Elastic settlement occurs immediately due to applied load without any change in the water content. Primary consolidation is a main component of settlement of fine-grained saturated soils having low permeability, because the excess pore water pressure dissipates with time. The secondary consolidation is creep of soils such as peats and soft organic clays under constant effective stress. The primary and secondary consolidation settlements can be several times greater than the elastic settlement in saturated fine-grained soils.

The consolidation settlement of a normally consolidated soil (s_c_) due to an increase in vertical stress (Δσ_v_) can be determined as;

1 $$s_{c} = \frac{{H_{0} }}{{1 + e_{0} }} \cdot C_{c} \cdot \log \left( {\frac{{\sigma_{v0}^{\prime } + \Delta \sigma_{v} }}{{\sigma_{v0}^{\prime } }}} \right)$$

where; C_c_ is the compression index; H_0_, e_0_ and $\sigma_{{{\text{v}}0}}^{{\prime }}$ are initial thickness, initial void ratio and average vertical effective stress of the soil layer, respectively.

If the final effective stress ($\sigma_{v0}^{\prime } + \Delta \sigma_{v}$) is less than the preconsolidation stress ($\sigma_{p}^{{\prime }}$) of the over-consolidated soil, then recompression index C_r_ can be used in place of compression index C_c_ in Eq. 1. When the final effective stress exceeds the preconsolidation stress, the settlement equation consists of two parts and both C_r_ and C_c_ must be used to calculate the consolidation settlement of over-consolidated soil as:

2 $$s_{c} = \frac{{H_{0} }}{{1 + e_{0} }} \cdot C_{r} \cdot \log \left( {\frac{{\sigma_{p}^{\prime } }}{{\sigma_{v0}^{\prime } }}} \right) + \frac{{H_{0} }}{{1 + e_{0} }} \cdot C_{c} \cdot \log \left( {\frac{{\sigma_{v0}^{\prime } + \Delta \sigma_{v} }}{{\sigma_{p}^{\prime } }}} \right)$$

**Fig. 1:**
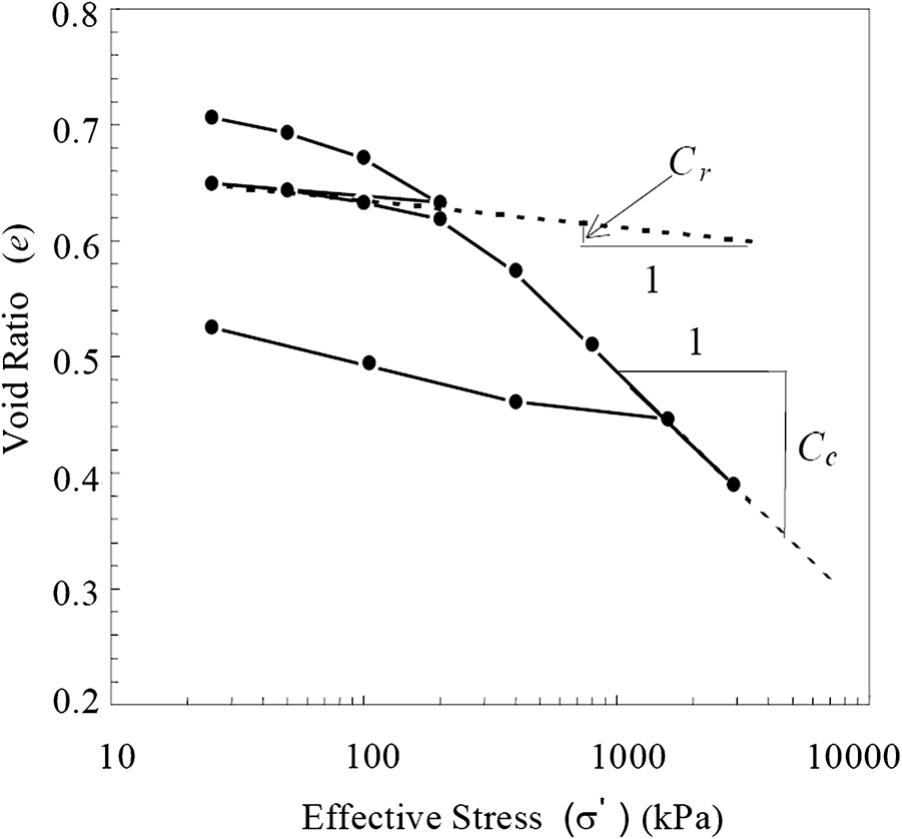
Definition of C_c_ and C_r_ from compression curve

The compressibility parameters such as compression index and recompression index are usually obtained by using the graphical analysis of compression curve in void ratio—effective stress (*e* − *log σ*) plots in Fig. 1. The slope of the straight-line portion of the virgin part of the compression curve on a semi-logarithmic plot is the compression index (C_c_) and the slope of the recompression or swelling curve is the recompression index (C_r_) as shown in Fig. 1.

## Database compilation

The database used in this study consist of 246 laboratory oedometer and index tests results of fine-grained soils obtained by the various geotechnical investigations in different locations in Turkey which belongs to the public agencies (State Hydraulic Works, General Directorate of Highways, Municipality) and private geotechnical companies. Soil parameters used in the database are the natural water content, liquid limit, plasticity index, initial void ratio, recompression index and compression index. The index and consolidation properties of soil samples were determined based on ASTM standard test methods. Almost all of the soil samples are classified according to USCS as low and high plasticity clay (CL–CH), and all of them are normally consolidated or lightly over-consolidated (OCR < 2.5). Statistical description of the input and output soil parameters in the database are shown in Table 1 and the values of the natural water content, liquid limit and plasticity index are given in percent.

**Table 1:** Descriptive statistics of parameters

	w_n_	LL	PI	e_0_	C_c_	C_r_
Least	16.3	26.3	9.0	0.460	0.070	0.011
Most	72.0	99.6	77.8	1.888	0.833	0.164
Mean	33.1	51.8	30.0	0.949	0.286	0.051
SD	11.3	14.4	12.9	0.313	0.150	0.031

The database that can be used for the development of regression-based new equations and comparison of the performance of existing regression-based empirical equations, involves a wide range of data as seen on the Table 1. However, the database was only created to determine the compression index and the recompression index from physical parameters of fine grained soils by using ANN in this study.

**Fig. 2:**
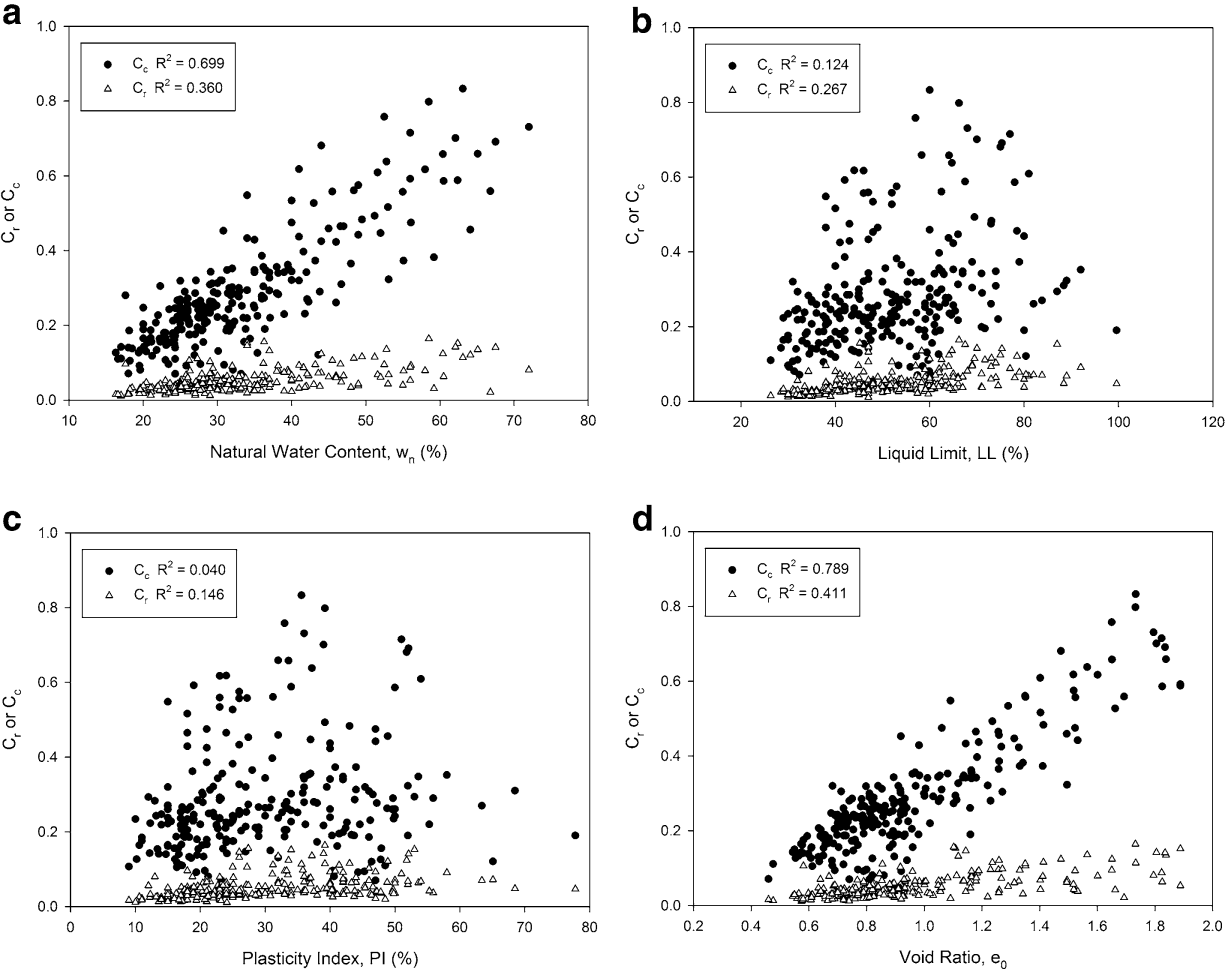
The relationships between compressibility and index parameters of the samples;** a** natural water content (w_n_) and C_r_ or C_c_,** b** liquid limit (LL) and Cr or Cc,** c** plasticity index (PI) and Cr or Cc,** d** void ratio (e_0_) and Cr or Cc

The compressibility parameters of the soils were supposed to be affected mainly by state parameters such as natural water content and initial void ratio, and intrinsic parameters such as liquid limit and plasticity index. The relationships between the compressibility parameters and index parameters of the soils used in this study are shown in Fig. 2. It is shown that the natural water content and the initial void ratio have more relatively linear correlations with the recompression–compression indexes than liquid limit and plasticity index of fine grained soils. In fully disturbed remoulded soil samples, the compressibility has a strong relationship with the intrinsic variables because the samples ideally lose all memory related to soil structure or stress history (Lee et al. 2015). However, the compressibility parameters of natural soils are particularly affected by the in situ state parameters such as sedimentation, deposition environments, stress history and natural soil conditions. The correlations between C_c_ and w_n_ or e_0_ in Fig. 2 have supported the idea.

## Artificial neural network (ANN)

**Fig. 3:**
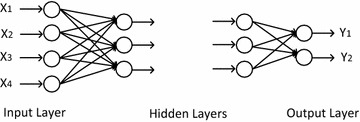
A sample structure of ANN

ANNs, created based on the biological neural networks, are calculating or operating systems which consist of large number of interconnected simple processors. Process elements of the ANNs are nonlinear circuits with nth degree named as cell. These circuits are called node too. Every node can have numerous input connections. However, there should be only one connection at their outputs. Output part is calculated depend on a selected mathematical model (Oztemel 2003). On the other hand, ANNs are divided into subsets which include neurons and named as layer. Input layer is a layer, have inputs come from external world to ANN. In this layer, process elements transfer information to the hidden layers as receiving from external world. Hidden layer is the layer where the information comes from the input layer. Incoming information from the input layer are processed in the hidden layer and forwarded to the output layer. The number of hidden layers can be changed according to the network structure. The increase in the number of neurons in the hidden layer boosts the complexity and calculation time. Nevertheless, this structure also enables the use of ANN in solving more complex problems. Output layer is the layer that produces outputs that correspond to the data from the input layer of the network by processing information from the hidden layer. The outputs generated in this layer are sent to the external world. Figure 3 shows a sample network.

The network is produced by the interconnection of the layers. There are three type of network in ANN. In feed-forward networks, the processor elements are decomposed into layers and flow of the information moves only one direction from the input layer to the output layer in these networks (Sagiroglu et al. 2003). In the ANNs connected in cascade, cells only receive information from the cells in the previous layer (MATLAB 2009). ANN with back propagation is widely used because of being useful and safe. The most important features of this type of ANN are being eligible for estimation, classification and to be useful in contains nonlinear structural models (Demuth et al. 2007). Also both feed forward and back propagation network structures can be described.

Basically learning methods in the ANN are divided into three groups. These are supervised learning which the training data is used for ANN’s training, unsupervised learning which is the set of weights of the connection of the mathematical relationship between the data without using any training kit and last one is reinforced learning which is a close method to supervised learning (Oz et al. 2002; MATLAB 2002). ANN model structure, network type and learning method used for the study are specified in the relevant section.

## Proposed artificial neural network model

In the ANN models, the available database is generally divided into three subsets: training, validation and testing sets. In this study, 70 % of the 246 samples (172 randomly selected data) for training, 15 % of the total data (37 randomly selected data) for validation and also 15 % of the database (37 randomly selected data) for testing were used to predict the compressibility parameters.

In order to evaluate the performance of the proposed ANN model, the correlation coefficient (R) and mean squared error (MSE) were used as statistical measures for comparison of the measured and predicted values. The correlation coefficient (R) and mean squared error (MSE) are given in Eqs. (3)–(4).

3 $$R = \frac{{\mathop \sum \nolimits_{i = 1}^{n} \left( {C_{i,m} - \overline{{C_{i,m} }} } \right)\left( {C_{i,p} - \overline{{C_{i,p} }} } \right)}}{{\sqrt {\mathop \sum \nolimits_{i = 1}^{n} \left( {C_{i,m} - \overline{{C_{i,m} }} } \right)^{2} \times \mathop \sum \nolimits_{i = 1}^{n} \left( {C_{i,p} - \overline{{C_{i,p} }} } \right)^{2} } }}$$

4 $$MSE = \frac{{\mathop \sum \nolimits_{i = 1}^{n} \left( {C_{i,m} - C_{i,p} } \right)^{2} }}{n}$$

where *C*_*i*,*m*_ and *C*_*i*,*p*_ are the measured and predicted output values; $\overline{C_{i,m}}$ and $\overline{C_{i,p}}$ are the averages of the measured and predicted output values, respectively. n is the number of sample.

**Fig. 4:**
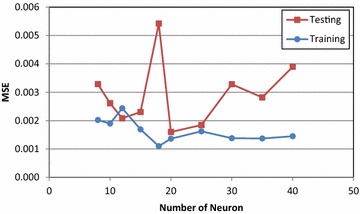
The optimization of the number of neurons in the hidden layer

The multilayer perceptron neural networks consist of three layers: input layer, hidden layer and output layer. Four basic soil parameters such as natural water content, liquid limit, plasticity index and initial void ratio were used as input parameters for the ANN model. The output layer consists of two neurons which are compression index and recompression index. In this model, ANN model using one hidden layer was preferred. A series of trial-and-error with different number of neuron between 8 and 40 were tried to find the optimum number of neurons in the hidden layer. At the end of these processes, MSE values for different number of neurons were obtained for the training and testing sets as shown in Fig. 4. The optimal architecture of the ANN model was determined based on the minimum mean square error and maximum correlation coefficient. The best performance was obtained from the ANN model with 20 neurons in the hidden layer. Therefore, the 20 neurons in the hidden layer can be considered as optimum value for the ANN model.

**Fig. 5:**
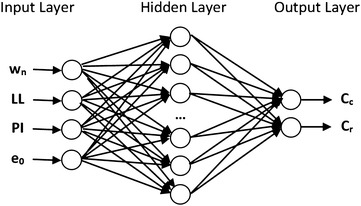
The architecture of the ANN model used for estimating C_c_ and C_r_

The feed-forward with back-propagation algorithm which is the most preferred algorithm (Rumelhart et al. 1986) in neural networks was used during the training stage. Standard Levenberg–Marquardt training function used as a learning algorithm in the developed ANN model. Additionally, a number of multilayer networks with different transfer functions for hidden and output layers were tried to predict the compressibility parameters. The most appropriate results for the network model were obtained from the sigmoid transfer function in the hidden layer and the linear transfer function in the output layer. The selected architecture of the ANN model used to predict the recompression and compression indexes of soil is shown in Fig. 5.

**Fig. 6:**
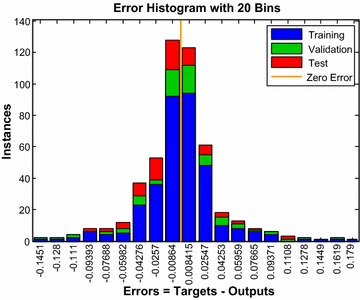
Error histogram

**Fig. 7:**
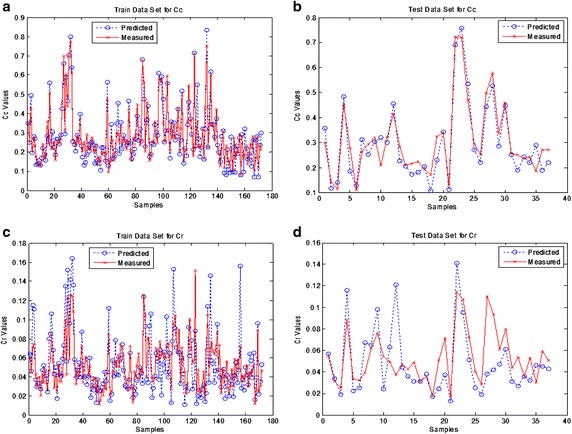
Simulation results;** a** training results of the C_c_ values for the proposed ANN model,** b** test results of the C_c_ values for the proposed ANN model,** c** training results of the C_r_ values for the proposed ANN model,** d** test results of the C_r_ values for the proposed ANN model

An error histogram can be examined for obtaining contrary data points at the ANN performance. Error histogram indicates that significant errors made on which estimated data and thus a neural network model in higher accuracy designing by purging incorrect data. Figure 6 shows that the error histogram of the obtained simulation results while there were 20 neurons in the hidden layer. The blue bars, the green bars and the red bars represent the training data, the validation data and the test data respectively at the error histogram. Considering the error histogram, the majority of the errors between the measured value and the predicted value are seen on between −0.04 and 0.04. The predicted and measured compression index and recompression index values for both training and test data have been shown in Fig. 7. It is seen that there are minor errors in the compression index data while the majority errors are in the recompression index data considering the differences between the measured and predicted values in the training and testing data.

**Fig. 8:**
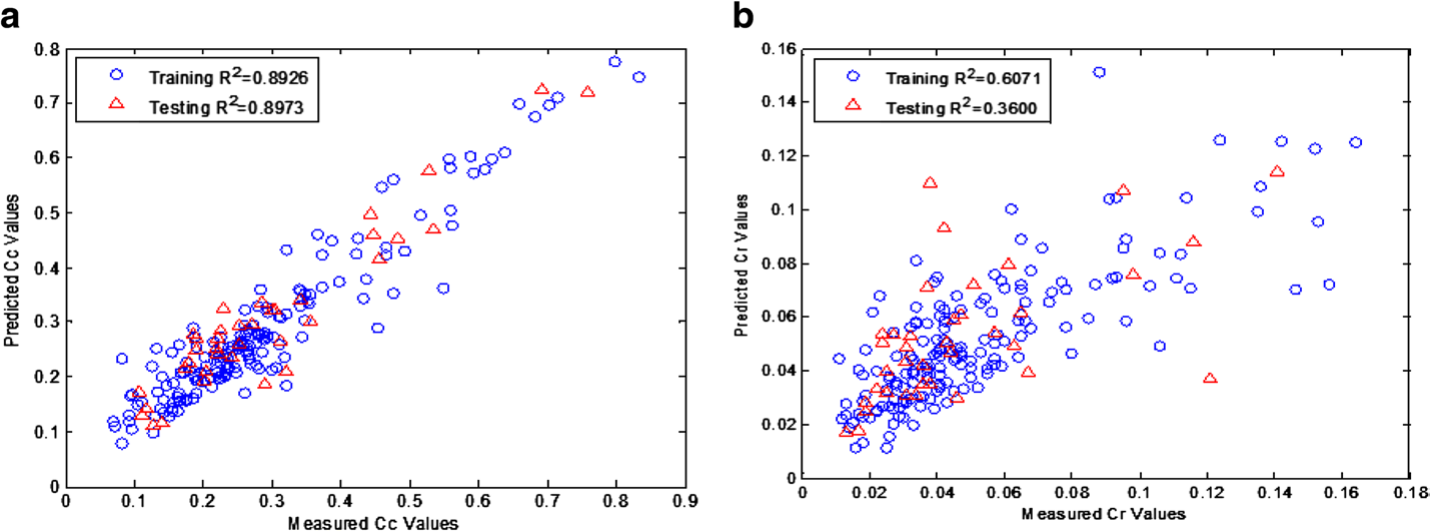
Comparison between the predicted and measured compression index (**a**) and recompression index (**b**)

Figure 8 shows the relationship between measured and predicted values obtained through the training and testing process. The calculated coefficients of determination (R^2^) for the compression index are 0.8926 and 0.8973 for training and testing stage, respectively. These results show that a quite close relationship between the measured values and the predicted values by ANN model. However, the coefficients of determination (R^2^) for the recompression index were calculated as 0.6071 and 0.3600 for training and testing, respectively. It is seen that the proposed ANN model obtained well correlation for the compression index compared with the recompression index.

## Discussions and conclusions

In this study, a neural network simulation practice has been made to predict the compression index and recompression index based on the geotechnical characteristics of different borehole data collected from Turkey. ANN is a powerful tool in predicting the consolidation parameters and more accurate results than the conventional methods are obtained. The previous studies based on this issue were generally focused on the predicting only compression index or recompression index by ANN. In this study, both the compression index and the recompression index are tried to predict on the combined ANN model structure. In the proposed ANN model, the input parameters are the soil properties such as the initial void ratio, the liquid limit, the natural water content and the plasticity index. The proposed model of the ANN results compared with the experimental values and the predicted compression index values were found close to the experimental values. However, the proposed ANN model did not show the same success for the recompression index data. This can be explained with the poor relationships between the recompression index and the physical properties in this study. The successful recompression index predictions with same input parameters should be difficult for the other data sets in similar ANN models. Nevertheless, the predicted compression index values using proposed ANN model are compatible with the measured compression index values as seen in this research.
